# Isocitrate Dehydrogenase-Wildtype Glioma Adapts Toward Mutant Phenotypes and Enhanced Therapy Sensitivity Under D-2-Hydroxyglutarate Exposure

**DOI:** 10.3390/biomedicines13071584

**Published:** 2025-06-28

**Authors:** Geraldine Rocha, Clara Francés-Gómez, Javier Megías, Lisandra Muñoz-Hidalgo, Pilar Casanova, Jose F. Haro-Estevez, Vicent Teruel-Martí, Daniel Monleón, Teresa San-Miguel

**Affiliations:** 1 Department of Pathology, University of Valencia, 46010 Valencia, Spain; geraldine.rocha@uv.es (G.R.); lisandra.munoz@uv.es (L.M.-H.); teresa.miguel@uv.es (T.S.-M.); 2 INCLIVA Biomedical Research Institute, 46010 Valencia, Spain; 3 Department of Anatomy, University of Valencia, 46010 Valencia, Spain; vicent.teruel@uv.es

**Keywords:** isocitrate dehydrogenase mutation, *IDH*, glioblastoma, astrocytoma, glioma, 2-hydroxyglutarate, orthotopic xenograft models, adjuvant radiotherapy, drug resistance in neoplasms

## Abstract

**Background/Objectives**: Isocitrate dehydrogenase (*IDH*) mutations are hallmark features in subsets of gliomas, producing the oncometabolite D-2-hydroxyglutarate (2HG). Although *IDH* mutations are associated with better clinical outcomes, their relationship with tumor progression is complex. This study aimed to investigate, in vitro and in vivo, the phenotypic consequences of *IDH* mutation and 2HG exposure in glioblastoma (GBM) under normoxic and hypoxic conditions and under temozolomide (TMZ) and radiation exposure. **Methods:** Experiments were conducted using *IDH*-wildtype (*IDH*-wt) and *IDH*-mutant (*IDH*-mut) glioma cell lines under controlled oxygen conditions. Functional assays included cell viability, cell cycle analysis, apoptosis profiling, migration, and surface marker expression via flow cytometry. Orthotopic xenografts were established in immunocompromised mice to assess in vivo tumor growth and morphology, followed by MRI and histological analysis. Treatments included TMZ, radiation, and 2HG at varying concentrations. Statistical analyses were performed using SPSS and RStudio. **Results:** *IDH*-wt cells exhibited faster proliferation and greater adaptability under hypoxia, while *IDH*-mut cells showed cell cycle arrest and limited growth. 2HG recapitulated *IDH*-mut features in *IDH*-wt cells, including increased apoptosis under TMZ, reduced proliferation, and altered CD24/CD44 expression. In vivo, *IDH*-wt tumors were larger and more infiltrative, while 2HG administration reduced tumor volume and promoted compact morphology. Notably, migration was initially similar across genotypes but increased in *IDH*-mut and 2HG-treated *IDH*-wt cells over time, though suppressed under therapeutic stress. **Conclusions:** *IDH* mutation and 2HG modulate glioma cell biology, including cell cycle dynamics, proliferation rates, migration, and apoptosis. While the *IDH* mutation and its metabolic product confer initial growth advantages, they enhance treatment sensitivity and reduce invasiveness, highlighting potential vulnerabilities for targeted therapy.

## 1. Introduction

Gliomas have historically been recognized as a heterogeneous group of primary central nervous system tumors with diverse biological behaviors and clinical outcomes. The integration of molecular biomarkers into their classification has transformed the field, particularly through the distinction between grade 4 adult-type diffuse gliomas: *IDH*-wildtype (*IDH*-wt) glioblastoma and *IDH*-mutant (*IDH*-mut) astrocytoma [1]. This molecular redefinition has improved prognostic accuracy [2,3], but its translation into tangible changes in therapeutic decision-making in neuro-oncology is still evolving [4,5,6].

*IDH*-wt glioblastomas are now diagnosed based on distinct molecular features, including *TERT* promoter mutations, EGFR amplification, or the combination of chromosome 7 gain with chromosome 10 loss. In contrast, grade 4 *IDH*-mut astrocytomas are classified using updated grading systems emphasizing alterations such *IDH1*/*2* mutations and *ATRX* loss [2,7]. Histologically, *IDH*-wt glioblastomas are associated with necrosis, microvascular proliferation, and aggressive growth patterns [8,9], whereas *IDH*-mut astrocytomas tend to display a more organized architecture and lower proliferation indices [8]. These differences have clear prognostic implications. *IDH*-mut astrocytomas are linked to longer survival compared to the poorer outcomes observed in *IDH*-wt glioblastoma [10]. This classification has provided clarity in a previously overlapping spectrum of high-grade gliomas and opened the door to biologically informed treatment strategies.

The identification of *IDH* mutations marked a critical inflection point in glioma research. These mutations were found in 73% of secondary glioblastomas but only in 3.7% of primary cases, revealing fundamental biological differences between subtypes [11]. Since then, *IDH* mutation status has become a stronger prognostic indicator than traditional histology [3,12]. Functionally, *IDH* mutations lead to the production of the so-called oncometabolite D-2-hydroxyglutarate (2HG), which disrupts DNA and histone demethylation, contributing to epigenetic dysregulation and tumorigenesis [13,14]. Tumors harboring these mutations display the glioma-CIMP phenotype, characterized by widespread DNA hypermethylation [15,16].

Although progress has been made, the precise role of 2HG in glioma biology remains unclear. While 2HG accumulation is linked to oncogenic processes resulting from *IDH* mutations found in astrocytomas and other cancers [17,18], in glioblastomas, this absence correlates with increased aggressiveness and therapy resistance [19], although the underlying mechanisms remain poorly understood. To explore this, we conducted in vitro experiments to determine whether exogenous 2HG could induce *IDH*-mut-like traits in *IDH*-wt glioma cells. We assessed changes in cellular behavior with and without 2HG, both under baseline conditions and during temozolomide (TMZ) and radiation treatment. Furthermore, we extended our investigation to an in vivo model, assessing tumor growth in mice after 2HG treatment to explore the potential sensitization of *IDH*-wt tumors. This study aims to clarify whether 2HG can modulate tumor cell phenotype and provide mechanistic insight into its functional role in glioma progression.

## 2. Materials and Methods

### 2.1. Cell Culture, Treatment Conditions, and Cell Growth Curve

In this study, U87MG (*IDH*-wt) and U87-IDHmut (*IDH*-mut) cell lines (ATCC, Manassas, VA, USA) were used and cultured under different experimental conditions, including normoxia (21% O_2_) and hypoxia (1% O_2_, 5% CO_2_, 94% N_2_), in a controlled chamber (BioSpherix, Parish, NY, USA). Experimental groups included untreated controls and treatments with TMZ at 25 μM (Sigma-Aldrich, Burlington, MA, USA), irradiation 2 Gy (X-Rad225XL; Precision X-Ray, North Branford, CT, USA), and 2HG at two different doses, 0.1 mM and 1 mM (Merck, Darmstadt, Germany). Cells were cultured in RPMI medium supplemented with 10% fetal bovine serum and 1% penicillin–streptomycin (Thermo Fisher Scientific, Waltham, MA, USA) and monitored daily using a phase-contrast microscope (Leica Microsystems, Wetzlar, Germany). For cell growth curve analysis, cells were seeded at a density of 100,000 cells per well in 6-well plates and maintained under normoxic or hypoxic conditions (1.5% O_2_) for ten days. Daily, cells were stained with Trypan Blue and counted using an automated cell counter (Bio-Rad Laboratories, Hercules, CA, USA).

### 2.2. Functional Characterization by Flow Cytometry

For cell death profiling, approximately 5 × 10^5^ cells were collected, centrifuged at 500× *g* for 5 min at 4 °C, and then resuspended in 200 μL PBS (Thermo Fisher Scientific, Waltham, MA, USA). Cells were stained with Apopxin Green (1.55 μL; Abcam, Cambridge, UK) for apoptotic detection, 7-AAD (0.81 μL; Abcam, Cambridge, UK) for necrotic identification, and CytoCalcein Violet 450 (0.92 μL; Abcam, Cambridge, UK) for viable cell assessment. Following a 15–30 min incubation at room temperature in light-protected conditions, samples were diluted in 300 μL assay buffer.

For cell cycle analysis, cells were fixed in cold 70% ethanol, washed, and then resuspended in 500 μL of a propidium iodide (PI) solution containing 0.1% RNase (BioLegend, San Diego, CA, USA). After overnight incubation at 2–8 °C in light-protected conditions, samples were processed to assess DNA content and cell cycle distribution, including G0/G1, S, and G2/M phases.

Proliferation tracking was performed via Carboxyfluorescein Succinimidyl Ester (CFSE) labeling. Cells were incubated with CFSE (1 μL of 5 mM; BioLegend, San Diego, CA, USA) for 20 min at room temperature in the dark, followed by quenching with complete culture medium (RPMI + 10% FBS), centrifugation, and resuspension in fresh medium. A subset of labeled cells was immediately analyzed to establish baseline fluorescence.

For phenotypic characterization, cells were stained with CD24, CD44, and CD45 (Life Technologies, Carlsbad, CA, USA) antibodies at optimized concentrations. Single-stained and Fluorescence Minus One (FMO) controls were included. Following a 15 min incubation at 4 °C in the dark, cells were washed, fixed, and resuspended in fixation buffer. For CD44^+^ cells, two gates were created to distinguish between CD44_High and CD44_Low subpopulations based on their relative expression levels. All experiments were performed on a BD LSRFortessa™ X-20 cytometer, and data were analyzed using FlowJo™ v10.8 Software (BD Biosciences, San Jose, CA, USA) [20].

### 2.3. Migration Assays

The migration assay was performed following seven days of treatment with the same doses of TMZ, irradiation, and 0.1 mM 2HG using culture inserts (Ibidi, Gräfelfing, Germany). A cell suspension (4 × 10^5^ cells/mL) was prepared in RPMI medium supplemented with 1% fetal bovine serum and 1% penicillin–streptomycin (Thermo Fisher Scientific, Waltham, MA, USA). After centrifuging to remove debris, 110 µL of this suspension was seeded per well. Cells were allowed to reach confluence over six days before inserts were removed to create a migration gap. Images were captured at 12, 24, 36, 48, and 72 h, as well as on day 6, using the LAS X Leica Application Suite X, version 3.7 (Leica Microsystems, Wetzlar, Germany). Image analysis was performed using Fiji, version 2.0.0-rc-69/1.52p (open-source, based on National Institutes of Health, Bethesda, MD, USA) [21], which was also used for morphological analyses, including the extraction of surface area, volume, Feret diameter, circularity, and roundness.

### 2.4. In Vivo Evaluation of Tumor Growth in an Orthotopic Xenograft Mouse Model

#### 2.4.1. Experimental Design

This study was approved by the Ethics Committee for Animal Welfare of the University of Valencia (code: 2022VSCPEA0172; 22 August 2022). All procedures complied with institutional and national regulations, followed the 3Rs [22], and adhered to ARRIVE guidelines [23]. A randomized, longitudinal, controlled, and blinded in vivo study was conducted to assess the effect of 2-hydroxyglutarate (2HG) on tumor growth in orthotopic xenografts using *IDH*-wt and *IDH*-mut glioma cells. Three groups were established: *IDH*-wt + 0.9% NaCl, *IDH*-wt + 2HG, and *IDH*-mut + 0.9% NaCl. The experimental unit was one cage with four mice. Four animals per group were used, totaling twelve. Sample size was calculated a priori using G*Power, version 3.1.9.7 (Heinrich Heine University, Düsseldorf, Germany), based on an in vitro effect size of 2.39.

Male J:NU ATHYM-Foxn1^nu/nu mice (Janvier Labs, Le Genest-Saint-Isle, France), aged 6 weeks and weighing 15–20 g, were used. These immunodeficient mice were chosen for their lack of T cells, enabling tumor growth without immune interference. Animals were housed for five weeks at UCIM (University of Valencia) under controlled conditions (12 h light/dark cycle, 22 ± 2 °C), with ad libitum access to food and water, and environmental enrichment. Inclusion criteria included the following: male sex, 6 weeks of age, weight between 15 and 30 g, normal hematology, and no abnormalities on CT. Two animals from the *IDH*-mut group were excluded from MR analysis due to a lack of tumor growth, yielding two animals in this group and four in the others. No exclusions were made due to pain or distress, according to the Mouse Grimace Scale [24]. Animals were randomly assigned to groups using Microsoft Excel, Microsoft 365 version (Microsoft Corporation, Redmond, WA, USA). Each group was housed in a separate cage with fixed positioning and individual identification to minimize bias. Blinding was applied to veterinary staff, imaging personnel, and data analysts, while the treatment administrator remained unblinded.

#### 2.4.2. Stereotactic Surgery for Orthotopic Xenotransplantation and Treatment

Stereotactic implantation of cells into the hippocampus was performed as described previously [25], simulating a microenvironment that resembles the human brain vasculature and structure. Before surgery, mice were acclimated for one week, weighed for monitoring, and screened via hematology and CT. All instruments were sterilized. Anesthesia was induced with 5% isoflurane and maintained at 1.5–2%, with oxygen at 0.5–0.8 L/min. Ophthalmic ointment was applied, and local anesthesia with 5% lidocaine was administered at the incision site. Perioperative analgesia included intraperitoneal injections of buprenorphine (0.01 mL/g), enrofloxacin (0.015 mL/g), and meloxicam (0.005 mL/g). Mice were placed in a stereotactic frame (Stoelting Co., Wood Dale, IL, USA), and a 1 cm incision was made to expose the skull. Coordinates for hippocampal injection (AP 2.0; ML 1.5 R) were determined from Bregma. A Hamilton syringe (Hamilton Company, Bonaduz, Switzerland) delivered 4 µL of tumor cell suspension (78,000 cells/µL) at 0.5 µL/min. After a 2 min pause, the needle was withdrawn slowly. Bone wax was applied, and the incision sutured. Postoperative recovery was individually monitored, with daily wound care and analgesics administered as needed. Body weight was tracked throughout. Treatment consisted of 0.1 mL of 2HG (27 mM) or 0.9% NaCl, administered intraperitoneally twice weekly, based on previous in vitro experiments. Corrective measures were in place for injection issues, and a clinical scoring system guided humane endpoints.

#### 2.4.3. Magnetic Resonance Imaging

Magnetic Resonance Imaging (MRI) was performed to monitor intratumoral alterations non-invasively. Imaging was carried out using a 3.0T MRS*DRYMAG 3017 scanner (MR-Solutions, Guildford, UK) following standardized acquisition protocols. Animals were anesthetized with isoflurane (3–4% for induction and 1–2.5% for maintenance), and physiological parameters, including body temperature and respiratory rate, were continuously monitored throughout the procedure. MRI sequences included T1-weighted, T2-weighted, and FLASH 3D, enabling the evaluation of tumor volume using 3D Slicer, version 5.2.2 (Brigham and Women’s Hospital, Boston, MA, USA). For contrast enhancement, Gadovist at 0.6 mmol/kg (Bayer, Leverkusen, Germany) was administered intraperitoneally prior to image acquisition.

### 2.5. Statistical Analysis

Statistical analyses were performed using IBM SPSS Statistics, version 27.0 (IBM Corp., Armonk, NY, USA) and RStudio, version 2023.12.0+402 (Posit Software, Boston, MA, USA). For quantitative data derived from cytometry assays (including proliferation, surface marker expression, and cell cycle analysis), cell migration assays, and in vivo tumor volume measurements, data normality was evaluated using the Kolmogorov–Smirnov test, and homogeneity of variances was assessed with Levene’s test. When assumptions were met, unpaired two-tailed Student’s *t*-tests were applied to compare two experimental groups, and one-way ANOVA followed by Bonferroni post hoc tests was used for comparisons involving more than two groups. When variance heterogeneity was detected, the Games–Howell test was employed. A significance threshold of α = 0.05 was adopted throughout. Data from growth curves and migration assays were analyzed using absolute values, while cytometric variables such as marker intensities and proliferation indices were normalized as required for comparability. Multivariate analyses, including Principal Component Analysis (PCA) and heatmaps, were conducted in R, version 4.3.2 (R Foundation for Statistical Computing, Vienna, Austria) following *z*-score standardization to ensure equal variable contribution. Visualizations were generated using ggplot2 and Complex Heatmap [26,27], and statistical significance in PCA groupings was inferred through cluster tendencies. Results were considered statistically significant at *p* < 0.05.

## 3. Results

### 3.1. Differential Cellular Behavior and Adaptive Responses Associated with IDH Mutation Status

#### 3.1.1. Differences in Proliferation and Sensitivity to Hypoxia Based on *IDH* Mutation Status

**Figure 1:**
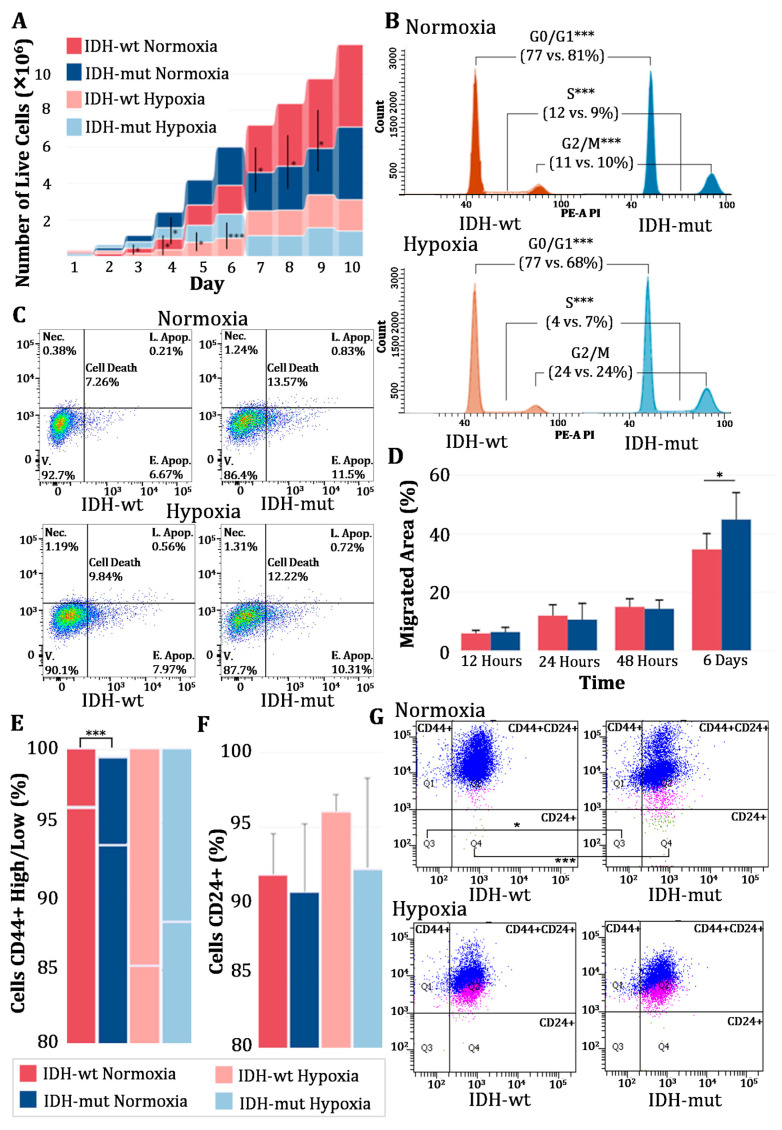
Differences between *IDH*-wt and *IDH*-mut cells under normoxic and hypoxic conditions. (**A**) Ribbon bar chart of the growth curve of *IDH*-wt and *IDH*-mut cells. (**B**) Cell cycle histogram of the distribution in *IDH*-wt and *IDH*-mut cells (x-axis: PE-A signal intensity; y-axis: number of events). (**C**) Gated dot plots of cell death in *IDH*-wt and *IDH*-mut cells. Quadrants indicate necrosis (Nec.), late apoptosis (L. Apop.), early apoptosis (E. Apop.), and viable cells (V.). Total cell death corresponds to the sum of necrosis and early and late apoptosis. Color intensity reflects event density in each region of the plot. (**D**) Bar chart of cell migration in *IDH*-wt and *IDH*-mut cells under normoxia. (**E**) Stacked bar chart of CD44^+^ marker expression in *IDH*-wt and *IDH*-mut cells. Lower bars represent CD44^+^ High and upper bars represent CD44^+^ Low populations. (**F**) Bar chart of CD24^+^ marker expression in *IDH*-wt and *IDH*-mut cells. (**G**) Gated dot plots of CD24 and CD44 co-expression profiles in *IDH*-wt and *IDH*-mut cells. Quadrants: Q1 = CD24^−^/CD44^+^, Q2 = CD24^+^/CD44^+^, Q3 = CD24^−^/CD44^−^, and Q4 = CD24^+^/CD44^−^. Asterisks indicate statistically significant differences between *IDH*-wt and *IDH*-mut under normoxic and hypoxic conditions (* *p* < 0.05, *** *p* < 0.001). *IDH*-wt: *IDH* wildtype; *IDH*-mut: *IDH* mutant.

*IDH*-wt cells exhibited a higher proliferative capacity than *IDH*-mut cells under normoxic conditions. After nine days in culture, the number of *IDH*-wt cells was significantly higher than that of *IDH*-mut cells (3.81 × 10^6^ vs. 2.53 × 10^6^; *p* = 0.02), evidence that the *IDH*-wt cells promote faster growth under normal oxygen conditions, as shown in Figure 1A.

However, hypoxia had a differential impact on the proliferation of both cell lines. Between days 3 and 6, the growth rate of *IDH*-mut cells was significantly higher than that of *IDH*-wt cells. By day 6, the number of *IDH*-wt cells reached only 9.73 × 10^5^ (*p* < 0.001), demonstrating greater sensitivity to oxygen depletion. In contrast, *IDH*-mut cells exhibited a greater adaptive capacity, with increased growth under hypoxia (1.32 × 10^6^ cells), indicating that the *IDH* mutation confers a proliferative advantage in conditions of low oxygen availability.

#### 3.1.2. Cell Cycle Modulation by *IDH* Mutation and the Hypoxic Microenvironment

Cell cycle analysis revealed fundamental differences between *IDH*-wt and *IDH*-mut cells (Figure 1B). Under normoxia, the *IDH* mutation promoted preferential arrest in the G0/G1 phase (*p* < 0.001), indicating a more restrictive control of the cell cycle. Conversely, *IDH*-wt cells exhibited higher proliferative activity, with a significant increase in the S and G2/M phases (*p* < 0.001), supporting their higher growth rate under these conditions.

Hypoxia altered the cell cycle distribution in both cell lines, doubling the proportion of cells in the G2/M phase compared to normoxia. In *IDH*-wt cells, the percentage of cells in G2/M increased from 10.77% to 24.06%, while in *IDH*-mut cells, it rose from 9.8% to 24.71%, with no statistically significant differences between *IDH*-mut and *IDH*-wt.

Additionally, hypoxia modulated the cell cycle in a mutation-specific manner. In *IDH*-mut cells, the proportion of cells in G0/G1 decreased substantially (from 81.5% under normoxia to 67.9% under hypoxia). Meanwhile, in *IDH*-wt cells, hypoxia reduced the proportion of cells in the S phase from 12.5% to 4.25%, with a greater impact than in *IDH*-mut cells (*p* < 0.001), showing a reduced replication capacity in this context.

#### 3.1.3. Differential Susceptibility to Cell Death Based on *IDH* Mutation Status Under Normoxia and Hypoxia

*IDH*-mut cells exhibited a higher susceptibility to cell death compared to *IDH*-wt cells under both normoxic and hypoxic conditions. Under normoxia, the proportion of cell death in *IDH*-mut cells was significantly higher (13.57 ± 0.4%) than in *IDH*-wt cells (7.26 ± 5.58%; *p* = 0.032). This trend persisted under hypoxia, where *IDH*-mut cells continued to show a higher cell death rate (12.22 ± 1.5% vs. 9.84 ± 1.44%; *p* = 0.031). Furthermore, hypoxia induced a general increase in cell death in both lines compared to normoxia, indicating that oxygen reduction acts as a stress factor affecting cell viability, with a more pronounced impact on *IDH*-mut cells (Figure 1C).

#### 3.1.4. *IDH*-Mutant Cells Exhibit Increased Migration over Time Under Normoxic Conditions

Under normoxia, the migration of both cell lines was comparable during the first 48 h. However, by day 6, *IDH*-mut cells exhibited significantly greater migration than *IDH*-wt cells (*p* = 0.05). At 12 h, the migrated area in *IDH*-mut cells was 6.28 ± 1.61%, while *IDH*-wt cells reached 5.82 ± 1.04% (*p* = 0.32). At 24 h, migration increased to 10.53 ± 5.55% in *IDH*-mut cells and 11.89 ± 3.68% in *IDH*-wt cells, with no significant differences. By 48 h, both groups displayed a further increase in migrated area, reaching 14.23 ± 2.97% in *IDH*-mut and 14.90 ± 2.77% in *IDH*-wt cells (*p* = 0.38). However, by day 6, *IDH*-mut cells demonstrated significantly greater migration (44.66 ± 9.20%) compared to *IDH*-wt cells (34.53 ± 5.42%, *p* = 0.05). These findings indicate that *IDH*-mut cells display higher long-term migratory capacity under basal conditions (Figure 1D).

#### 3.1.5. Differential Expression of Cellular Plasticity Markers Based on *IDH* Mutation Status

Our analysis revealed that *IDH*-mut cells displayed distinct subpopulation distributions based on CD24 and CD44 expression (Figure 1E,F). Specifically, we observed a higher proportion of CD24^+^/CD44^−^ and CD44^+^/CD24^−^ cell populations within the *IDH*-mut group compared to *IDH*-wt cells. Under normoxia, the proportion of cells with these phenotypes was significantly higher in *IDH*-mut cells (1.27% and 24.07%, respectively) compared to *IDH*-wt cells (0.17% and 17.2%, respectively; *p* < 0.001; *p* = 0.106). Additionally, *IDH*-wt cells generally exhibited a higher proportion of CD44^+^ cells than *IDH*-mut cells (99.23% vs. 98.5%; *p* = 0.001). Under hypoxia, the proportion of CD24^+^/CD44^−^ and CD44^+^/CD24^−^ cells in *IDH*-mut decreased (0.27% and 22.93%, respectively), while in *IDH*-wt cells, the values remained similar to those observed under normoxia (0.167 ± 0.047%; *p* = 0.150) (Figure 1G). Thus, hypoxia induces differential phenotypic changes that depend on the *IDH* mutational status.

### 3.2. Differential Cellular Responses to Radiotherapy and Temozolomide in *IDH*-Mutant and *IDH*-Wildtype Cells

#### 3.2.1. Modulation of Cell Cycle Progression and Apoptotic Response Under Treatments

**Figure 2:**
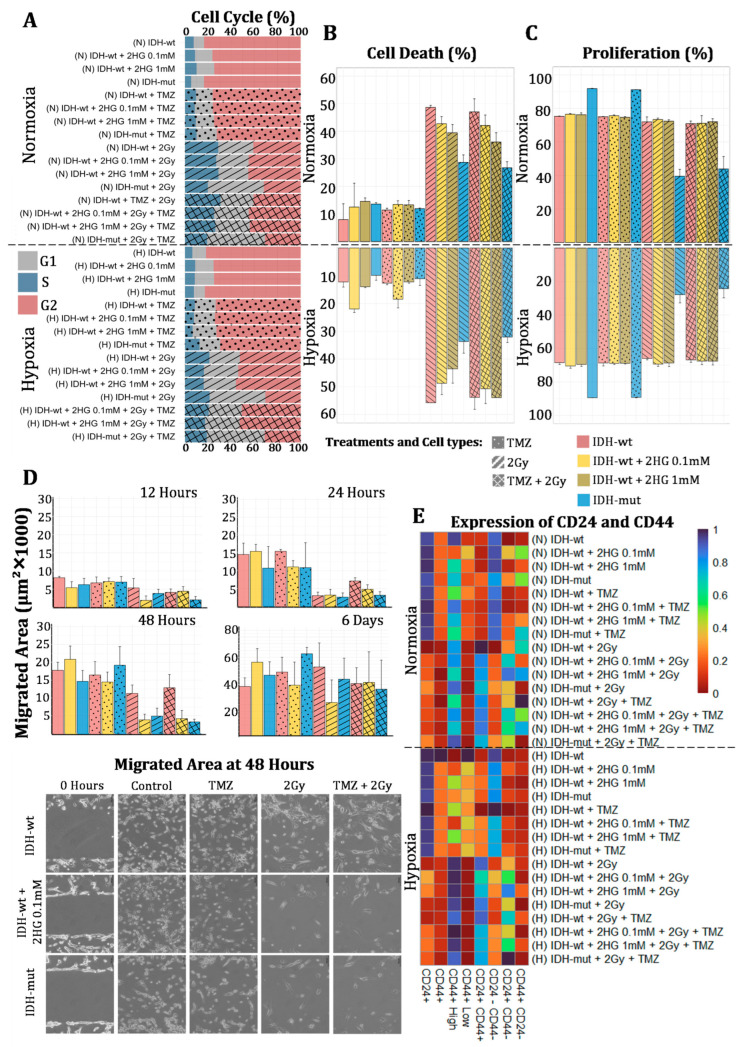
Differences between *IDH*-wt, *IDH*-wt + 2HG (0.1 mM), *IDH*-wt + 2HG (1 mM), and *IDH*-mut cells in response to TMZ (temozolomide), 2 Gy radiation, and the combination (TMZ + 2 Gy) under normoxic and hypoxic conditions. (**A**) Stacked bar chart of cell cycle distribution (G1, S, and G2 phases). Cell groups are indicated by color, and treatment conditions by patterns. (**B**) Bar chart of total cell death (sum of early apoptosis, late apoptosis, and necrosis). Cell groups are represented by color, and treatments by patterns. (**C**) Bar chart of cell proliferation across treatment conditions. Cell groups are represented by color, and treatments by patterns. (**D**) Bar chart of cell migration at 12, 24, 48 h, and 6 days. Below, representative images of wound healing at 48 h are shown. (**E**) Heatmap of CD44^+^ and CD24^+^ marker expression (*z*-score scale). Blue indicates higher expression values, and red indicates lower values. N: Normoxia, H: Hypoxia, *IDH*-wt: *IDH* wildtype; *IDH*-mut: *IDH* mutant; 2HG: 2-hydroxyglutarate; 2Gy: 2 Gray dose of ionizing X-ray radiation.

Treatment with TMZ and radiation altered cell cycle profiles in both *IDH*-mut and *IDH*-wt cell lines. Under normoxic conditions, radiation induced an accumulation of cells in the G2/M phase, which was significantly higher in *IDH*-mut cells (*p* = 0.002). This effect was enhanced when combined with TMZ, resulting in a further increase in G2/M-phase cells in the *IDH*-mut group (*p* < 0.001) (Figure 2A). Under hypoxia, the accumulation in the G2/M phase was more pronounced in both lines, with a marked increase in *IDH*-mut cells.

Although both lines showed changes in cell cycle distribution, apoptosis levels differed significantly. Under normoxia, radiation reduced cell viability in both cell types, but early apoptotic rates were higher in *IDH*-wt cells (43.53%) compared to *IDH*-mut cells (25.9%, *p* < 0.001). Total cell death was also significantly elevated in *IDH*-wt cells (48.66%) versus *IDH*-mut cells (28.71%, *p* < 0.001) (Figure 2B).

Combined TMZ and radiation treatment under hypoxia resulted in 30.4% total apoptosis in *IDH*-mut cells, significantly lower than in *IDH*-wt cells (51.12%, *p* < 0.001). Under these conditions, *IDH*-mut cells also maintained higher proliferative activity.

#### 3.2.2. Increased Migration and Proliferation in *IDH*-Mutant Cells and Their Sensitivity to Treatment

*IDH*-mut cells exhibited enhanced proliferative and migratory capacities compared to *IDH*-wt cells under normoxic and hypoxic conditions. *IDH*-mut cells displayed significantly higher proliferation rates (*p* < 0.001), and although both lines exhibited similar migration at early time points, *IDH*-mut cells demonstrated significantly greater migration by day 6 (*p* = 0.05) (Figure 2C,D).

Interestingly, treatment with TMZ unexpectedly enhanced the migratory capacity of both cell lines, with *IDH*-mut cells displaying significantly higher migration at later time points compared to *IDH*-wt cells (*p* = 0.03). In contrast, radiation therapy alone markedly suppressed migration in both cell types, with a more substantial inhibitory effect observed in *IDH*-mut cells (*p* < 0.001). Notably, the combined treatment with TMZ and radiation further reduced migration, particularly in *IDH*-mut cells at early time points, resulting in a significantly greater decrease in motility relative to *IDH*-wt cells (*p* < 0.001).

Similarly, proliferation patterns mirrored these findings. Under normoxia, radiation significantly decreased proliferation in both cell lines, with a greater impact on *IDH*-mut cells (*p* < 0.001). The combination of TMZ and radiation further reduced proliferation, reinforcing the idea that *IDH*-mut cells are more vulnerable to DNA-damaging therapies. Under hypoxia, *IDH*-mut cells maintained high proliferation rates similar to those observed under normoxia, while *IDH*-wt cells exhibited a moderate reduction. The resistance of *IDH*-wt cells to TMZ was evident in both normoxia and hypoxia, as this treatment did not induce significant changes in their proliferation. In contrast, in *IDH*-mut cells, the combination of TMZ and radiation resulted in a marked reduction in proliferation (*p* < 0.001), with the *IDH*-mut cells being particularly susceptible to therapeutic interventions under hypoxic conditions.

These results show that *IDH*-mut cells display higher proliferative and migratory activity in baseline conditions, but their motility and growth are more strongly inhibited by radiation and combined therapy.

#### 3.2.3. Differential Expression of CD44 and CD24 in Response to Treatment

Treatment-modulated expression of the surface markers CD44 and CD24 revealed distinct patterns between *IDH*-wt and *IDH*-mut cells. Under normoxic conditions, TMZ significantly decreased the proportion of CD44^+^ cells in *IDH*-wt cultures (*p* = 0.008), while no significant change was observed in *IDH*-mut cells (Figure 2E). Additionally, *IDH*-mut cells exhibited a higher percentage of CD24^+^/CD44^−^ cells compared to *IDH*-wt (*p* = 0.003). Under hypoxia, CD44^+^ expression increased significantly in *IDH*-wt cells after combined TMZ and radiation treatment (*p* = 0.016). In contrast, radiation alone reduced CD44^+^ cell proportions in both lines, with a stronger effect in *IDH*-mut cells (*p* < 0.001).

CD24 expression was significantly decreased following radiation in both *IDH*-mut and *IDH*-wt cells, regardless of oxygen availability. However, a higher proportion of CD24^+^ cells persisted in *IDH*-mut populations (*p* = 0.001). The combination of TMZ and radiation further reduced the CD44^+^/CD24^+^ subpopulation, with a more pronounced reduction in *IDH*-mut cells (*p* < 0.001). An increase in CD44^+^/CD24^−^ cells was also observed following radiation in both cell lines, and this effect was significantly more prominent in *IDH*-mut cells under hypoxia (25.88%) compared to *IDH*-wt (10.23%, *p* = 0.029).

These findings indicate that *IDH*-mut cells retain a higher CD24^+^ fraction and exhibit a greater shift toward a CD44^+^/CD24^−^ phenotype after treatment, particularly under hypoxic conditions.

### 3.3. Impact of D-2-Hydroxyglutarate on *IDH*-wt Cells

#### 3.3.1. Redefinition of the Cell Cycle: Increased G0/G1 Phase and Transition Toward the *IDH*-Mutant Phenotype

Treatment with 2HG in *IDH*-wt cells subjected to radiotherapy and TMZ induced significant alterations in cell cycle distribution, shifting the behavior toward that observed in *IDH*-mut cells. Under normoxic conditions, the addition of 2HG to irradiated cells resulted in an increased fraction of cells in the G0/G1 phase, reaching 33.50 ± 0.57% (*p* = 0.007) at 0.1 mM and 32.87 ± 1.44% (*p* = 0.012) in the presence of TMZ—values that exceeded those of the control (Figure 2A).

Under hypoxic conditions, exposure to 2HG further elevated the proportion of cells in G0/G1, registering 43.63 ± 2.41% (*p* = 0.009) at 1 mM and 47.77 ± 1.67% (*p* = 0.015) at 0.1 mM. This increase was accompanied by a significant reduction in the S phase (13.83 ± 1.04% vs. 17.50 ± 1.80%, *p* = 0.006 at 1 mM and 14.53 ± 1.096%, *p* = 0.015 at 0.1 mM) and an increase in the G2/M phase (23.83 ± 0.309%, *p* = 0.003 at 1 mM; 26.267 ± 1.744%, *p* = 0.002 at 0.1 mM). Moreover, the combination of radiotherapy and TMZ at 1 mM led to a reduction in the S phase to 15 ± 0.829% (*p* = 0.023) and an increase in the G2/M phase (25.77 ± 0.52% vs. 21.83 ± 0.95%, *p* < 0.001).

#### 3.3.2. Enhanced Apoptosis and Modulation of Cell Viability

The effect of 2HG on cell death mechanisms was evaluated based on apoptosis, necrosis, and overall cell viability. Under normoxia, exposure to 2HG at 1 mM significantly increased early apoptosis (13.73 ± 0.90% vs. 6.67 ± 5.25%, *p* = 0.019) and total apoptosis (14.25 ± 1.04% vs. 6.89 ± 5.50%, *p* = 0.020), resulting in decreased cell viability (85.43 ± 1.02% vs. 92.77 ± 5.57%, *p* = 0.021). A similar pattern was observed at 0.1 mM, where early apoptosis reached 13.01 ± 1.15% (*p* = 0.036) and total apoptosis 13.43 ± 1.07% (*p* = 0.036), with viability decreasing to 86.57 ± 1.09% (*p* = 0.036) (Figure 2B).

The combination of 2HG with TMZ further amplified these effects, increasing early apoptosis (12.27 ± 1.12% vs. 6.67 ± 5.25%, *p* = 0.041 at 1 mM and 12.33 ± 1.12%, *p* = 0.040 at 0.1 mM) and total apoptosis (12.92 ± 1.20% vs. 6.89 ± 5.50%, *p* = 0.038 at 1 mM and 13.01 ± 1.15%, *p* = 0.036 at 0.1 mM), consequently reducing viability. Under normoxic radiotherapy, administration of 2HG at 1 mM decreased necrosis (1.08 ± 0.55 vs. 2.08 ± 0.45, *p* = 0.015) and early apoptosis (35.33 ± 2.46 vs. 43.53 ± 0.58, *p* < 0.001), which was associated with an increase in viability (60.57 ± 2.45 vs. 51.30 ± 0.64, *p* < 0.001) and a reduction in total cell death (39.43 ± 2.45 vs. 48.66 ± 0.60, *p* < 0.001). At 0.1 mM, a reduction in early apoptosis (37.33 ± 2.74 vs. 43.53 ± 0.58, *p* = 0.002) was observed, along with a slight increase in viability (57.30 ± 2.10 vs. 51.30 ± 0.64, *p* = 0.001).

Under hypoxic conditions, 2HG at 1 mM increased early apoptosis (12.60 ± 0.16 vs. 10.31 ± 1.42, *p* = 0.009) and total apoptosis (13.29 ± 0.16 vs. 11.03 ± 1.53, *p* = 0.013), while reducing necrosis (0.65 ± 0.33 vs. 1.19 ± 0.10, *p* = 0.010). At 0.1 mM, the pro-apoptotic effect was even more pronounced, with early apoptosis doubling (21.00 ± 0.86 vs. 10.31 ± 1.42, *p* < 0.001) and total apoptosis similarly increasing (21.64 ± 0.95 vs. 11.03 ± 1.53, *p* < 0.001). When combined with TMZ under hypoxia, 2HG at 1 mM reduced necrosis (0.91 ± 0.18 vs. 1.19 ± 0.10, *p* = 0.015) without significant changes in apoptosis, whereas at 0.1 mM, marked increases in early (17.00 ± 2.67 vs. 10.31 ± 1.42, *p* = 0.002) and total apoptosis (17.86 ± 2.73 vs. 11.03 ± 1.53, *p* = 0.002) were observed.

Under hypoxic radiotherapy, 2HG at 1 mM significantly reduced necrosis (1.05 ± 0.38 vs. 2.98 ± 0.53, *p* = 0.001) and apoptosis (early: 40.43 ± 3.84 vs. 47.10 ± 5.41, *p* = 0.046; total: 42.49 ± 4.20 vs. 52.76 ± 5.46, *p* = 0.012), while at 0.1 mM, there was a reduction in late apoptosis (4.31 ± 0.56 vs. 5.66 ± 1.00, *p* = 0.028) and in total cell death (45.64 ± 3.95 vs. 52.76 ± 5.46, *p* = 0.040) coupled with an increase in the fraction of viable cells (51.23 ± 3.34 vs. 44.27 ± 4.91, *p* = 0.029). The combination of 2HG and TMZ at 1 mM under hypoxic radiotherapy further reduced necrosis (0.89 ± 0.17 vs. 2.98 ± 0.53, *p* < 0.001) and late apoptosis (4.51 ± 0.28 vs. 5.66 ± 1.00, *p* = 0.034).

Taken together, 2HG increased apoptosis and reduced viability under baseline and TMZ-treated conditions, whereas under radiotherapy, particularly in hypoxia, it was associated with decreased necrosis and total cell death.

#### 3.3.3. Adjustment of Cellular Proliferation: Modulation of CFSE and Convergence Toward the *IDH*-Mutant Profile

Cellular proliferation, assessed via CFSE percentage, was also modulated by 2HG treatment. Under normoxic conditions, treatment with 2HG at 0.1 mM reduced CFSE to 23.47 ± 0.31% (*p* < 0.001), while at 1 mM, the CFSE value was 23.8 ± 0.94% (*p* = 0.05). The combination of 2HG at 1 mM with TMZ increased CFSE to 25.30 ± 0.34% (*p* = 0.01), indicating a reduction in proliferation, though not to the same extent as observed in *IDH*-mut cells (Figure 2C).

Under hypoxia, 2HG at 0.1 mM decreased CFSE to 29.51 ± 1.09% (*p* = 0.02), and when combined with TMZ, CFSE markedly dropped to 1.68 ± 0.08% (*p* = 0.03). In the context of radiotherapy under hypoxia, *IDH*-wt cells exhibited a CFSE of 34.00 ± 0.67% compared to 72.13 ± 4.02% in *IDH*-mut cells (*p* < 0.001); the addition of 2HG at 1 mM and 0.1 mM reduced CFSE to 31.28 ± 1.74% (*p* = 0.01) and 30.62 ± 1.09% (*p* = 0.001), respectively.

#### 3.3.4. Inhibition of Migration and Morphological Transformation: Impact on Migratory Area and Cellular Parameters

2HG exposure in *IDH*-wt cells under normoxia modulated migratory behavior and morphology, with effects varying according to treatment. While the combination with radiotherapy reduced migration and altered morphology, 2HG alone showed a tendency to enhance migration over time.

In cells treated with 2HG alone, the migratory area remained similar to controls during the first 48 h (5390 µm^2^ at 12 h, 15,443 µm^2^ at 24 h, and 20,815 µm^2^ at 48 h). By day 6, it increased to 56,390 µm^2^, significantly higher than that of the control group (38,463 µm^2^, *p* = 0.029). A similar increase was observed with the addition of TMZ at 24 h (11,070 µm^2^, *p* = 0.012) (Figure 1D).

In contrast, combining 2HG with radiotherapy significantly reduced the migratory area at 48 h compared to radiotherapy alone (3855 µm^2^ vs. 11,265 µm^2^, *p* = 0.003). The triple combination with radiotherapy and TMZ further decreased migration at 24 h (4802 µm^2^ vs. 7125 µm^2^, *p* = 0.028) and 48 h (4198 µm^2^ vs. 12,853 µm^2^, *p* = 0.011).

Morphological analysis showed reduced circularity after 2HG treatment (0.250 ± 0.010 vs. 0.316 ± 0.024, *p* = 0.001), along with decreases in Feret diameter and cell circumference at 24 and 48 h (*p* < 0.001 and *p* = 0.010). Combined with TMZ, 2HG further reduced the circumference (0.291 ± 0.012, *p* < 0.001) and increased signal intensity at day 6 (270.176 ± 71.159), similar to values observed in *IDH*-mut cells treated with TMZ (119.448, *p* = 0.018).

#### 3.3.5. Phenotypic Reprogramming: Transition Toward a Less Differentiated State

Analysis of phenotypic markers demonstrated that 2HG induces a reprogramming toward a less differentiated state in *IDH*-wt cells. Under normoxic conditions, the addition of 2HG at 1 mM increased total CD24 expression (95.30 ± 0.28 vs. 91.80 ± 2.26 in control, *p* = 0.011) and the CD24^+^CD44^−^ population (0.47 ± 0.09 vs. 0.17 ± 0.04, *p* = 0.001). A similar increase was observed at 0.1 mM (94.77 ± 2.25, *p* = 0.056), and in the presence of TMZ, the total CD24 positive cell population reached 95.10 ± 1.92 (*p* = 0.034). The fraction of CD44^−^CD24^−^ cells increased with 2HG at 0.1 mM (0.43 ± 0.28 vs. 0.06 ± 0.04, *p* = 0.023) and 1 mM (0.13 ± 0.04, *p* = 0.046).

The dot plot of CD44 positive cells revealed two distinct subpopulations. They were analyzed separately and designated as CD44^+^ High and CD44^+^ Low, based on their relative CD44 expression levels (Figure 2E). Under normoxia with 2HG at 1 mM, a reduction in High_CD44 (96.03 ± 2.03 vs. 92.60 ± 0.57, *p* = 0.009) was accompanied by an increase in Low_CD44 (7.90 ± 0.54 vs. 4.20 ± 2.26, *p* = 0.010), an effect that was more pronounced with 2HG at 0.1 mM (High_CD44: 96.03 ± 2.03 vs. 87.37 ± 4.80, *p* = 0.008; Low_CD44: 13.00 ± 4.71 vs. 4.20 ± 2.26, *p* = 0.008) and was comparable to the effect observed with TMZ (91.27 ± 2.85, *p* = 0.017).

Under normoxic radiotherapy, 2HG at 1 mM increased total CD24 (29.23 ± 4.37 vs. 13.53 ± 6.84, *p* = 0.004) and the CD44^+^CD24^+^ population (34.13 ± 3.58 vs. 20.17 ± 6.91, *p* = 0.006), with similar changes observed at 0.1 mM (total CD24: 28.00 ± 5.51, *p* = 0.008; CD44^+^CD24^+^: 32.66 ± 3.73, *p* = 0.010).

Under hypoxic conditions, 2HG at 1 mM significantly reduced total CD24 (93.23 ± 2.39 vs. 96.07 ± 0.95, *p* = 0.035), a trend that persisted when combined with TMZ (93.50 ± 1.34, *p* = 0.010 at 1 mM and 93.20 ± 2.26, *p* = 0.029 at 0.1 mM). The CD44^+^CD24^−^ population increased with 2HG at 1 mM (25.73 ± 8.30 vs. 14.13 ± 4.36, *p* = 0.024) and with the combination of 2HG + TMZ at 1 mM (26.90 ± 5.17, *p* = 0.005) and 0.1 mM (27.60 ± 7.65, *p* = 0.011), while the CD44^+^CD24^+^ fraction decreased with 2HG at 1 mM (74.07 ± 8.25 vs. 85.57 ± 4.36, *p* = 0.024) and with 2HG + TMZ at 1 mM (72.67 ± 5.46, *p* = 0.005) and 0.1 mM (72.07 ± 7.54, *p* = 0.011).

Under hypoxic radiotherapy, 2HG at 1 mM reduced the CD44^+^CD24^−^ population (59.83 ± 0.89 vs. 70.47 ± 2.45, *p* < 0.001) and increased CD44^+^CD24^+^ (38.73 ± 1.54 vs. 28.87 ± 2.37, *p* < 0.001); similar effects were observed with 2HG at 0.1 mM (CD44^+^CD24^−^: 54.30 ± 1.70, *p* < 0.001; CD44^+^CD24^+^: 44.96 ± 1.69, *p* < 0.001). Additionally, the combination of 2HG and TMZ under hypoxic radiotherapy further decreased the CD44^+^CD24^−^ population while increasing CD44^+^CD24^+^, consolidating a phenotypic profile that closely resembles that of *IDH*-mut cells.

In summary, the results indicate that 2HG exposure led to notable changes in the differentiation markers of *IDH*-wt cells, showing phenotypic reprogramming. Under normoxia, 2HG increased total CD24 and both the CD24^+^CD44^−^ and CD44^−^CD24^−^ populations, and it altered CD44^+^ distribution by reducing High_CD44 and increasing Low_CD44. Under normoxic radiotherapy, 2HG also elevated total CD24 and the CD44^+^CD24^+^ population. Conversely, under hypoxia, 2HG decreased total CD24 and the CD44^+^CD24^+^ fraction, while increasing the CD44^+^CD24^−^ population. When combined with radiotherapy under hypoxia, 2HG and TMZ reinforced this phenotypic modification, further reducing CD44^+^CD24^−^ and increasing CD44^+^CD24^+^.

#### 3.3.6. Multivariate Analysis of Experimental Conditions

**Figure 3:**
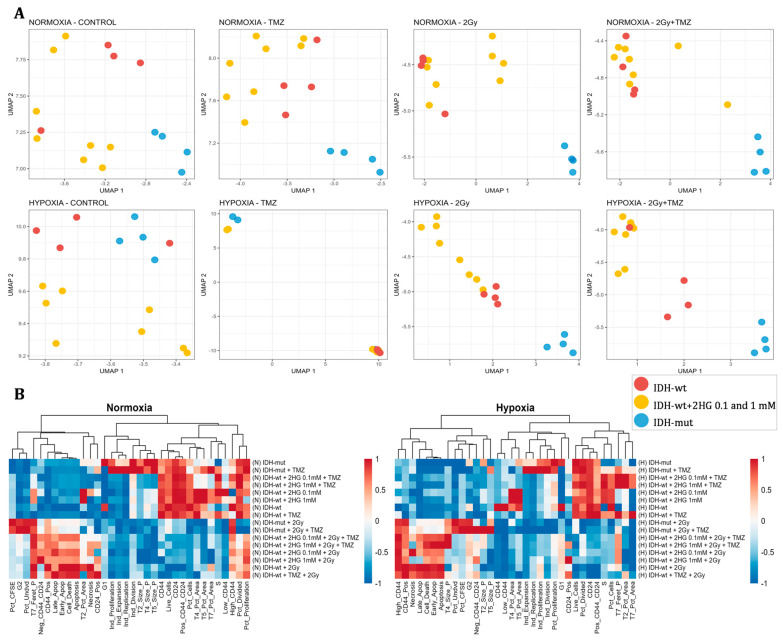
Uniform Manifold Approximation and Projection (UMAP) and hierarchical clustering heatmaps of experimental conditions. (**A**) UMAP plots of experimental conditions under normoxia (top row) and hypoxia (bottom row) across different treatments. Each point represents a sample. Plot columns correspond to treatment conditions: control, TMZ, 2 Gy radiation, and the combination (2 Gy + TMZ). (**B**) Hierarchical clustering heatmaps of samples under normoxic (right) and hypoxic (left) conditions. Red indicates higher values; blue indicates lower values. N: Normoxia, H: Hypoxia, *IDH*-wt: *IDH* wildtype; *IDH*-mut: *IDH* mutant; 2HG: 2-hydroxyglutarate; 2Gy: 2 Gray dose of ionizing X-ray radiation.

To evaluate whether the experimental variables could differentiate sample groups, a UMAP dimensionality reduction was performed using all measured parameters. As shown in Figure 3A, samples were segregated according to *IDH* status, oxygen conditions, and treatment. Clustering was particularly evident in groups treated with 2 Gy or 2 Gy + TMZ, especially under hypoxia, where the effect of 2HG treatment on *IDH*-wt cells was observed by the clear separation of this group from *IDH*-wt cells without 2HG, despite undergoing radiotherapy or combined therapy. In contrast, control and TMZ-only groups showed more dispersed distributions. Hierarchical clustering and heatmaps (Figure 3B) further supported these groupings, revealing consistent patterns within treatments and highlighting differences between normoxic and hypoxic conditions.

### 3.4. D-2-Hydroxyglutarate Modulates Tumor Growth and Morphology, Mimicking IDH Mutation Effects In Vivo

To validate whether the in vitro effects of 2HG translate to an in vivo context, we conducted a pilot study in which xenografts were grown in immunodeficient mice. In this model, we analyzed the impact of 2HG on tumor growth and morphology. *IDH*-wt tumors exhibited the largest volumes, averaging 20.73 ± 9.19 mm^3^, with a mean Feret diameter of 5.57 ± 1.11 mm and a surface area of 61.41 ± 20.06 mm^2^. These tumors displayed an elongated morphology, with a roundness of 0.59 ± 0.03 and flatness of 1.27 ± 0.21. The voxel count averaged 4415.50 ± 1958.42.

Exposure to 2HG in *IDH*-wt tumors led to a marked reduction in tumor volume (12.75 ± 8.79 mm^3^) and Feret diameter (4.12 ± 1.07 mm), accompanied by a decrease in surface area (35.82 ± 20.05 mm^2^) in comparison with *IDH*-wt mice. Notably, 2HG-treated tumors exhibited a shift toward a more rounded phenotype (roundness = 0.72 ± 0.07, *p* = 0.049 vs. *IDH*-wt), alongside decreased flatness (1.10 ± 0.06) and elongation (1.39 ± 0.24). The number of voxels also dropped to 2715.00 ± 1873.14.

**Figure 4:**
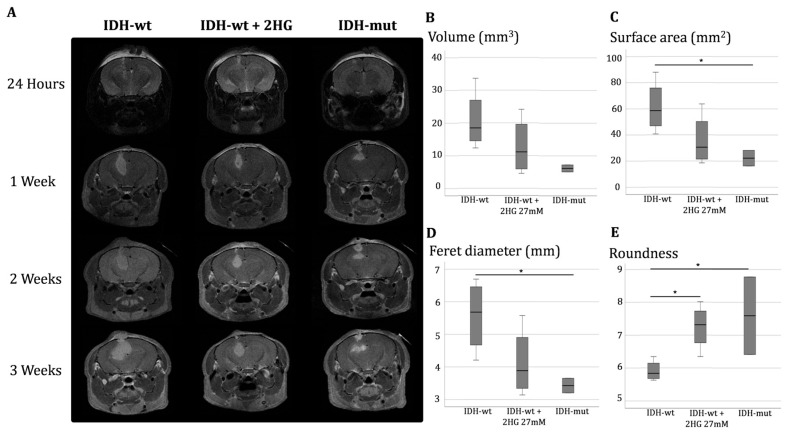
Longitudinal MRI assessment and quantification of tumor growth and morphology in *IDH*-wt, *IDH*-wt + 2HG (27 mM), and *IDH*-mut mouse groups. (**A**) Representative T2-weighted MRI images at 24 h and T1-weighted MRI images at 1, 2, and 3 weeks post-implantation in *IDH*-wt, *IDH*-wt + 2HG, and *IDH*-mut groups. (**B**) Quantification of tumor volume (mm^3^) at 3 weeks. (**C**) Tumor surface area measurements (mm^2^) at 3 weeks. (**D**) Feret diameter (mm) analysis of tumor size at 3 weeks. (**E**) Tumor roundness index at 3 weeks. Black lines inside the boxes indicate medians. Asterisks indicate statistically significant differences between groups (* *p* < 0.05). *IDH*-wt: *IDH* wildtype; *IDH*-mut: *IDH* mutant; 2HG: D-2-hydroxyglutarate.

*IDH*-mut tumors displayed the most compact and spherical architecture in comparison with *IDH*-wt mice, with a mean volume of 6.17 ± 1.52 mm^3^ and Feret diameter of 3.43 ± 0.32 mm. Surface area was substantially lower in this group (22.28 ± 8.41 mm^2^, *p* = 0.051 vs. *IDH*-wt), while roundness was the highest among the groups (0.76 ± 0.17). Flatness (1.10 ± 0.02) and elongation (1.25 ± 0.01) were also reduced. The voxel count was lowest at 1314.50 ± 323.15. These quantitative imaging analyses revealed striking differences in tumor volume and morphology across the experimental groups, highlighting the influence of *IDH* mutation and 2HG exposure on tumor architecture (Figure 4).

## 4. Discussion

### 4.1. IDH Status Influences Tumor Growth and Adaptability

In this work, we present a study of the impact of *IDH* mutation and its metabolic product, 2HG, on the growth and behavior of *IDH*-wt GBM cells under various environmental conditions, with particular focus on cellular stress responses. For the first time to our knowledge, we report an increase in the radio- and chemo-sensitivity of *IDH*-wt glioma cells after 2HG exposure. We extensively characterize the cellular and biological response of wildtype and *IDH*-mutated U87 cells under different conditions and treatments and irradiation exposure. Our results strongly suggest a potential role of 2HG as a modulator of tumor progression, adaptability, and therapy sensitivity in gliomas.

Although *IDH* mutations are associated with better clinical outcomes, their relationship with tumor progression is complex. Our data revealed significant differences in the growth dynamics and migration capabilities of *IDH*-wt and *IDH*-mut cells. On the one hand, *IDH*-wt cells grew faster than *IDH*-mut cells under both normoxic and hypoxic conditions. While both cell types showed reduced growth under hypoxia, *IDH*-wt cells adapted better, maintaining a higher proliferation rate over time in low-oxygen environments.

This difference in adaptability may be related to cell cycle regulation and differential proliferative profiles. CFSE-based flow cytometry also revealed distinct proliferation dynamics. Under normoxia, *IDH*-mutant cells proliferated more rapidly than *IDH*-wt cells. Under hypoxia, proliferation decreased in both genotypes, but *IDH*-mut cells still showed higher rates. These proliferation profiles align well with previous reports of reduced HIF-1α stabilization in *IDH*-mut gliomas and increased glycolytic adaptation in *IDH*-wt tumors [28,29]. On the other hand, our orthotopic glioblastoma xenograft model reveals that *IDH*-wt tumors grew significantly faster than *IDH*-mut tumors, with volumes increasing over three-fold, larger Feret diameters and surface areas, and more elongated morphology. All these findings indicate a more aggressive and infiltrative phenotype.

### 4.2. 2HG Modulates Therapy Sensitivity and Phenotypic Traits

TMZ and radiation treatment induced cell cycle changes in both genotypes, with *IDH*-mut cells exhibiting a stronger G2/M arrest. Despite this, apoptotic responses were lower in *IDH*-mut cells, particularly under hypoxia, where *IDH*-wt cells showed significantly higher apoptosis. *IDH*-mut cells also showed stronger proliferation decreases after TMZ and radiation exposure. These results align with clinical data indicating superior radiochemotherapy responses in *IDH1*-mut gliomas [30,31]. Collectively, all these data suggest a potential role of *IDH* mutation in the interaction between glioma cells and the tumor microenvironment. Our results support the idea that *IDH* mutation and 2HG regulate a delicate balance between cell cycle progression, stress response, and cell death, influenced by external stressors like hypoxia and therapy.

*IDH* mutations lead to 2HG accumulation [32], altering glutaminolysis and lipid metabolism and supporting short-term proliferation. Other research links *IDH1* mutations to *mTOR* pathway activation [33], promoting survival signaling, while 2HG modulates epigenetic regulators and provides early growth advantages [34,35]. However, this advantage diminishes over time due to impaired metabolic flexibility and stress responses in *IDH*-mut cells [36].

In our study, exogenous 2HG administration to *IDH*-wt cells mimicked some phenotypic features of *IDH*-mut cells. On the one hand, 2HG increased early and total apoptosis, especially when combined with TMZ, leading to reduced viability. On the other hand, 2HG increased proliferation in *IDH*-wt cells, especially under hypoxia. Paradoxically, under radiotherapy, 2HG protected cells, reducing apoptosis and necrosis while enhancing survival. This dual response was also observed under hypoxia, where 2HG promoted apoptosis in some contexts, particularly with TMZ, but reduced cell death when combined with radiation, particularly at lower doses.

Although migration assays show only marginal differences between wildtype and *IDH*-mutant glioma cells, under radiation or radiation plus TMZ, 2HG suppressed *IDH*-wt migration. Importantly, intraperitoneal administration of 2HG to *IDH*-wt xenografted mice led to significant tumor shrinkage, evidenced by smaller Feret diameters and surface areas. The 2HG-treated tumors were more compact, with increased roundness and reduced elongation, implying less invasive growth, consistent our findings in U87 cells. Our results support the notion that *IDH*-mut cells are more radiosensitive and show that 2HG induces similar vulnerability in *IDH*-wt cells both in vitro and in vivo.

### 4.3. Phenotypic Reprogramming via 2HG Exposure

Beyond its impact on proliferation and therapy response, 2HG also induced notable phenotypic reprogramming in *IDH*-wt glioma cells. In our model, exogenous 2HG altered the expression of CD24 and CD44 in *IDH*-wt glioma cells. Under normoxia, 2HG increased total CD24 levels, expanded the CD24^+^/CD44^−^ subpopulation, and reduced CD44 High expression, resembling the *IDH*-mut phenotype. This agrees with prior findings showing that 2HG upregulates CD24 by promoting DNA hypomethylation and decreasing repressive marks like H3K27me3 [37]. CD24 is linked to less aggressive behavior and better prognosis, while CD44 is associated with mesenchymal, therapy-resistant states [38]. The observed shift towards higher CD24 and lower CD44 suggests a transition to a less invasive, more therapy-sensitive phenotype.

Importantly, our aim was not to induce stable phenotypic reprogramming, but rather to determine whether transient exposure to 2HG could sensitize *IDH*-wt glioma cells to conventional therapies. Given the aggressive nature and rapid progression of GBM, even short-term modulation prior to standard treatment may hold clinical value.

Overall, our findings highlight the dual and context-dependent role of 2HG as both an oncometabolite and a modulator of therapy response and cellular phenotype. These effects are consistent with the extensive epigenetic remodeling observed in *IDH*-mutant gliomas, where 2HG promotes DNA and histone hypermethylation by inhibiting α-KG–dependent demethylases. Both clinical and preclinical studies have confirmed these alterations in patients and model systems [15,39]. Our findings align with these observations, supporting the hypothesis that 2HG is not only a byproduct but also an active modulator of glioma cell behavior.

### 4.4. Limitations and Future Directions

Our study has certain limitations. First, the in vitro experiments were conducted using a single *IDH1*-mut glioma cell line generated via CRISPR-Cas9, which provided a consistent genetic background and minimized variability due to inter-patient differences. However, this also restricted the breadth of our observations to a single model. Future studies will be necessary to validate these findings across additional *IDH*-mut cell lines and patient-derived models.

Second, although we incorporated an orthotopic xenograft model in mice to address this limitation and better approximate the tumor microenvironment, allowing us to study tumor behavior within the brain in a more physiologically relevant context, this model does not capture immune–tumor interactions. The use of immunodeficient mice allowed us to isolate the intrinsic effects of 2HG on glioma biology without confounding immune influences. However, it also precludes evaluation of potential immunomodulatory roles of 2HG, since immune system interactions are critical regulators of tumor progression and therapy response [40]. Future studies using immunocompetent or humanized models will be necessary to assess whether 2HG influences glioma immunogenicity or tumor–immune dynamics.

## 5. Conclusions

In conclusion, our results demonstrate that *IDH* mutations and the accumulation of their oncometabolite 2HG profoundly alter glioblastoma cell behavior across multiple biological levels, including proliferation, cell cycle regulation, apoptosis, migration, and phenotypic plasticity. While *IDH*-mut cells may exhibit early proliferative activity and enhanced basal migration under favorable conditions, they show impaired adaptability to stress, particularly under hypoxia or in response to cytotoxic treatments. This vulnerability is characterized by a reduced capacity to activate apoptotic pathways despite G2/M arrest, as well as a context-dependent modulation of survival and migration in the presence of 2HG. Conversely, 2HG exposure in *IDH*-wt cells often recapitulates features of the mutant phenotype, altering cell cycle dynamics, increasing radiosensitivity, shifting CD24/CD44 expression, and sensitizing cells to TMZ.

These findings underscore the complex role of 2HG in modulating glioma cell behavior, particularly under stress conditions, and suggest that its context-dependent effects could be harnessed to enhance the efficacy of current treatments. By mimicking key features of the *IDH*-mut phenotype, such as increased radiosensitivity and altered expression of therapeutic targets, 2HG or its downstream pathways may offer promising avenues for the development of novel, targeted therapies in *IDH*-wt gliomas.

## Data Availability

In support of research reproducibility and open science, the data supporting the findings of this study, including processed datasets and detailed methodologies, are available from the corresponding author upon reasonable request. The data are not publicly available due to institutional policy and project-related confidentiality.

## Abbreviations

The following abbreviations are used in this manuscript:

*IDH*	Isocitrate Dehydrogenase
*IDH*-wt	*IDH* Wildtype
*IDH*-mut	*IDH* Mutant
TMZ	Temozolomide
2HG	D-2-hydroxyglutarate
2Gy	Radiation
CFSE	Carboxyfluorescein Succinimidyl Ester
MRI	Magnetic Resonance Imaging
N	Normoxia
H	Hypoxia
R	Radiation

## References

[1] Louis D.N., Perry A., Wesseling P., Brat D.J., Cree I.A., Figarella-Branger D., Hawkins C., Ng H.K., Pfister S.M., Reifenberger G. (2021). The 2021 WHO Classification of Tumors of the Central Nervous System: A Summary. Neuro-Oncology.

[2] Brat D.J., Aldape K., Colman H., Holland E.C., Louis D.N., Jenkins R.B., Kleinschmidt-DeMasters B.K., Perry A., Reifenberger G., Stupp R. (2018). cIMPACT-NOW Update 3: Recommended Diagnostic Criteria for “Diffuse Astrocytic Glioma, *IDH*-Wildtype, with Molecular Features of Glioblastoma, WHO Grade IV”. Acta Neuropathol..

[3] Hartmann C., Hentschel B., Wick W., Capper D., Felsberg J., Simon M., Westphal M., Schackert G., Meyermann R., Pietsch T. (2010). Patients with *IDH1* Wild Type Anaplastic Astrocytomas Exhibit Worse Prognosis than *IDH1*-Mutated Glioblastomas, and *IDH1* Mutation Status Accounts for the Unfavorable Prognostic Effect of Higher Age: Implications for Classification of Gliomas. Acta Neuropathol..

[4] Stupp R., Mason W.P., van den Bent M.J., Weller M., Fisher B., Taphoorn M.J.B., Belanger K., Brandes A.A., Marosi C., Bogdahn U. (2005). Radiotherapy plus Concomitant and Adjuvant Temozolomide for Glioblastoma. N. Engl. J. Med..

[5] Valdebenito S., D’Amico D., Eugenin E. (2019). Novel Approaches for Glioblastoma Treatment: Focus on Tumor Heterogeneity, Treatment Resistance, and Computational Tools. Cancer Rep..

[6] Alshiekh Nasany R., de la Fuente M.I. (2023). Therapies for *IDH*-Mutant Gliomas. Curr. Neurol. Neurosci. Rep..

[7] Komori T. (2022). Grading of Adult Diffuse Gliomas According to the 2021 WHO Classification of Tumors of the Central Nervous System. Lab. Investig..

[8] Christians A., Adel-Horowski A., Banan R., Lehmann U., Bartels S., Behling F., Barrantes-Freer A., Stadelmann C., Rohde V., Stockhammer F. (2019). The Prognostic Role of *IDH* Mutations in Homogeneously Treated Patients with Anaplastic Astrocytomas and Glioblastomas. Acta Neuropathol. Commun..

[9] Shirahata M., Ono T., Stichel D., Schrimpf D., Reuss D.E., Sahm F., Koelsche C., Wefers A., Reinhardt A., Huang K. (2018). Novel, Improved Grading System(S) for *IDH*-Mutant Astrocytic Gliomas. Acta Neuropathol..

[10] Christians A., Banan R., Stockhammer F., Hartmann C. (2019). PATH-11. The Prognostic Role of *IDH* Mutations in Homogeneously Treated Patients with Malignant Diffuse Astrocytomas. Neuro-Oncology.

[11] Nobusawa S., Watanabe T., Kleihues P., Ohgaki H. (2009). *IDH1* Mutations as Molecular Signature and Predictive Factor of Secondary Glioblastomas. Clin. Cancer Res..

[12] Levallet G., Creveuil C., Bekaert L., Péres E., Planchard G., Lecot-Cotigny S., Guillamo J.-S., Emery E., Zalcman G., Lechapt-Zalcman E. (2019). Promoter Hypermethylation of Genes Encoding for RASSF/Hippo Pathway Members Reveals Specific Alteration Pattern in Diffuse Gliomas. J. Mol. Diagn..

[13] Grassian A.R., Parker S.J., Davidson S.M., Divakaruni A.S., Green C.R., Zhang X., Slocum K.L., Pu M., Lin F., Vickers C. (2014). *IDH1* Mutations Alter Citric Acid Cycle Metabolism and Increase Dependence on Oxidative Mitochondrial Metabolism. Cancer Res..

[14] Borger D.R., Tanabe K.K., Fan K.C., Lopez H.U., Fantin V.R., Straley K.S., Schenkein D.P., Hezel A.F., Ancukiewicz M., Liebman H.M. (2012). Frequent Mutation of Isocitrate Dehydrogenase (*IDH*)1 and *IDH2* in Cholangiocarcinoma Identified Through Broad-Based Tumor Genotyping. Oncologist.

[15] Turcan S., Rohle D., Goenka A., Walsh L.A., Fang F., Yilmaz E., Campos C., Fabius A.W.M., Lu C., Ward P.S. (2012). *IDH1* Mutation Is Sufficient to Establish the Glioma Hypermethylator Phenotype. Nature.

[16] Lu C., Ward P.S., Kapoor G.S., Rohle D., Turcan S., Abdel-Wahab O., Edwards C.R., Khanin R., Figueroa M.E., Melnick A. (2012). *IDH* Mutation Impairs Histone Demethylation and Results in a Block to Cell Differentiation. Nature.

[17] Dang L., White D.W., Gross S., Bennett B.D., Bittinger M.A., Driggers E.M., Fantin V.R., Jang H.G., Jin S., Keenan M.C. (2009). Cancer-Associated *IDH1* Mutations Produce 2-Hydroxyglutarate Author Information R132H Mutant *IDH1* Structure Files Are Deposited in the Protein Data Bank under Accession Code 3INM. Nature.

[18] Figueroa M.E., Abdel-Wahab O., Lu C., Ward P.S., Patel J., Shih A., Li Y., Bhagwat N., Vasanthakumar A., Fernandez H.F. (2010). Leukemic *IDH1* and *IDH2* Mutations Result in a Hypermethylation Phenotype, Disrupt TET2 Function, and Impair Hematopoietic Differentiation. Cancer Cell.

[19] Hasselblatt M., Jaber M., Reuss D., Grauer O., Bibo A., Terwey S., Schick U., Ebel H., Niederstadt T., Stummer W. (2018). Diffuse Astrocytoma, *IDH*-Wildtype: A Dissolving Diagnosis. J. Neuropathol. Exp. Neurol..

[20] BD Biosciences (2023). FlowJo^TM^ Software.

[21] Schindelin J., Arganda-Carreras I., Frise E., Kaynig V., Longair M., Pietzsch T., Preibisch S., Rueden C., Saalfeld S., Schmid B. (2012). Fiji: An Open-Source Platform for Biological-Image Analysis. Nat. Methods.

[22] Russell W., Burch R. (1960). The Principles of Humane Experimental Technique. Med. J. Aust..

[23] Kilkenny C., Browne W.J., Cuthill I.C., Emerson M., Altman D.G. (2010). Improving Bioscience Research Reporting: The ARRIVE Guidelines for Reporting Animal Research. PLoS Biol..

[24] Langford D.J., Bailey A.L., Chanda M.L., Clarke S.E., Drummond T.E., Echols S., Glick S., Ingrao J., Klassen-Ross T., LaCroix-Fralish M.L. (2010). Coding of Facial Expressions of Pain in the Laboratory Mouse. Nat. Methods.

[25] Baumann B.C., Dorsey J.F., Benci J.L., Joh D.Y., Kao G.D. (2012). Stereotactic Intracranial Implantation and In Vivo Bioluminescent Imaging of Tumor Xenografts in a Mouse Model System of Glioblastoma Multiforme. J. Vis. Exp. JoVE.

[26] Wickham H. (2016). ggplot2: Elegant Graphics for Data Analysis.

[27] Gu Z., Eils R., Schlesner M. (2016). Complex Heatmaps Reveal Patterns and Correlations in Multidimensional Genomic Data. Bioinformatics.

[28] McAfee D., Moyer M., Queen J., Mortazavi A., Boddeti U., Bachani M., Zaghloul K., Ksendzovsky A. (2023). Differential Metabolic Alterations in *IDH1* Mutant vs. Wildtype Glioma Cells Promote Epileptogenesis through Distinctive Mechanisms. Front. Cell. Neurosci..

[29] Zhao S., Lin Y., Xu W., Jiang W., Zhai Z., Wang P., Yu W., Li Z., Gong L., Peng Y. (2009). Glioma-Derived Mutations in *IDH1* Dominantly Inhibit *IDH1* Catalytic Activity and Induce HIF-1alpha. Science.

[30] Weller J., Katzendobler S., Blobner J., Thiele F., Becker H., Quach S., Egensperger R., Niyazi M., Suchorska B., Thon N. (2022). Limited Efficacy of Temozolomide Alone for Astrocytoma, *IDH*-Mutant, CNS WHO Grades 2 or 3. J. Neurooncol..

[31] Tran A.N., Lai A., Li S., Pope W.B., Teixeira S., Harris R.J., Woodworth D.C., Nghiemphu P.L., Cloughesy T.F., Ellingson B.M. (2013). Increased Sensitivity to Radiochemotherapy in *IDH1* Mutant Glioblastoma as Demonstrated by Serial Quantitative MR Volumetry. Neuro-Oncology.

[32] Waitkus M.S., Pirozzi C.J., Moure C.J., Diplas B.H., Hansen L.J., Carpenter A.B., Yang R., Wang Z., Ingram B.O., Karoly E.D. (2017). Adaptive Evolution of the GDH2 Allosteric Domain Promotes Gliomagenesis by Resolving IDH1R132H Induced Metabolic Liabilities. Cancer Res..

[33] Avsar T., Kose T.B., Oksal M.D., Turan G., Kilic T. (2022). *IDH1* Mutation Activates mTOR Signaling Pathway, Promotes Cell Proliferation and Invasion in Glioma Cells. Mol. Biol. Rep..

[34] Reitman Z.J., Jin G., Karoly E.D., Spasojevic I., Yang J., Kinzler K.W., He Y., Bigner D.D., Vogelstein B., Yan H. (2011). Profiling the Effects of Isocitrate Dehydrogenase 1 and 2 Mutations on the Cellular Metabolome. Proc. Natl. Acad. Sci. USA.

[35] Garrett M., Fujii Y., Osaka N., Ito D., Hirota Y., Sasaki A.T., Debinski W. (2021). Emerging Roles of Wild-Type and Mutant *IDH1* in Growth, Metabolism and Therapeutics of Glioma. Gliomas.

[36] Han S., Liu Y., Cai S.J., Qian M., Ding J., Larion M., Gilbert M.R., Yang C. (2020). *IDH* Mutation in Glioma: Molecular Mechanisms and Potential Therapeutic Targets. Br. J. Cancer.

[37] Tiburcio P.D.B., Locke M.C., Bhaskara S., Chandrasekharan M.B., Huang L.E. (2020). The Neural Stem-Cell Marker CD24 Is Specifically Upregulated in *IDH*-Mutant Glioma. Transl. Oncol..

[38] Bhat K.P.L., Balasubramaniyan V., Vaillant B., Ezhilarasan R., Hummelink K., Hollingsworth F., Wani K., Heathcock L., James J.D., Goodman L.D. (2013). Mesenchymal Differentiation Mediated by NF-κB Promotes Radiation Resistance in Glioblastoma. Cancer Cell.

[39] Huang L.E. (2019). Friend or Foe—*IDH1* Mutations in Glioma 10 Years on. Carcinogenesis.

[40] Hanahan D. (2022). Hallmarks of Cancer: New Dimensions. Cancer Discov..

